# S100a9 deficiency accelerates MDS-associated tumor escape via PD-1/PD-L1 overexpression: S100a9 deficiency accelerates tumor escape

**DOI:** 10.3724/abbs.2023015

**Published:** 2023-02-21

**Authors:** Roujia Wang, Youshan Zhao, Zijuan Li, Juan Guo, Sida Zhao, Luxi Song, Dong Wu, Lan Wang, Chunkang Chang

**Affiliations:** 1 Department of Hematology Shanghai Jiao Tong University Affiliated Sixth People’s Hospital Shanghai 200233 China; 2 CAS Key Laboratory of Tissue Microenvironment and Tumor Shanghai Institute of Nutrition and Health Shanghai Institutes for Biological Sciences University of Chinese Academy of Sciences Chinese Academy of Sciences Shanghai 200031 China

**Keywords:** MDS, S100a9, tumor escape

## Abstract

In recent studies, the tolerable safety profile and positive bone marrow (BM) response suggest a beneficial use of anti-PD-1 agents in the treatment of Myelodysplastic Syndromes (MDS), but the underlying mechanism is still unknown. MDS is mainly characterized by ineffective hematopoiesis, which may contribute to inflammatory signaling or immune dysfunction. Our previous studies focused on inflammatory signaling, and the results showed that S100a9 expression was higher in low-risk MDS and lower in high-risk MDS. In this study, we combine the inflammatory signaling and immune dysfunction. SKM-1 cells and K562 cells co-cultured with S100a9 acquire apoptotic features. Moreover, we confirm the inhibitory effect of S100a9 on PD-1/PD-L1. Importantly, PD-1/PD-L1 blockade and S100a9 can both activate the PI3K/AKT/mTOR signaling pathway. The cytotoxicity is higher in lower-risk MDS-lymphocytes than in high-risk MDS-lymphocytes, and S100a9 partially rescues the exhausted cytotoxicity in lymphocytes. Our study demonstrates that S100a9 may inhibit MDS-associated tumor escape via PD-1/PD-L1 blockade through PI3K/AKT/mTOR signaling pathway activation. Our findings indicate the possible mechanisms by which anti-PD-1 agents may contribute to the treatment of MDS. These insights may provide mutation-specific treatment as a supplementary therapy for MDS patients with high-risk mutations, such as TP53, N-RAS or other complex mutations.

## Introduction

The bone marrow microenvironment plays a key role in the pathogenesis of Myelodysplastic Syndromes (MDS). The cytotoxic effects of extracellular S100a8/a9 have been observed in human cancer cell lines [1]. Although these studies strongly indicate that S100a8/a9 elicit powerful anti-tumor responses, their functions in hematological malignancies remain poorly understood [2]. Binding of S100A9 to Toll-like receptor 4 (TLR4) promotes activation of the p38 mitogen-activated protein kinase, extracellular signal-regulated kinases 1 and 2, and Jun N-terminal kinase signaling pathways, leading to myelomonocytic and monocytic AML cell differentiation [3]. We previously demonstrated that S100a9 is highly expressed in lower-risk Myelodysplastic Syndromes-bone marrow-mononuclear cells (MDS-BM-MNCs) and expressed at low levels in higher-risk MDS-BM-MNCs [4].

In cancers, CD8 ^+^ T cells upregulate the expression of inhibitory costimulatory molecule, mediated negative signal transduction, resulting in dysfunction and apoptosis in CD8 ^+^ T cells, which are then described as exhausted CD8 ^+^ T cells [ 5‒ 8] . Programmed cell death protein 1 (PD-1) has been shown to be expressed on exhausted T cells and to be a major mechanism of immune escape that malignancies take advantage of to evade destruction [ 9, 10] . More importantly, Cheng *et al*. [11] found that S100a9 plays a critical role in the induction of PD-1/programmed cell death ligand 1 (PD-L1) surface receptor expression on hematopoietic stem and progenitor cells (HSPCs) and myeloid-derived suppressor cells (MDSCs). Therefore, in this study we aimed to explore the role of S100a9 in tumor escape and to identify its potential mechanisms.

## Materials and Methods

### Study participants

Patients were diagnosed with MDS in accordance with the Vienna diagnostic criteria 2017 [12]. Fourteen patients with MDS and three healthy controls were included in this study; their characteristics are detailed in Supplementary Table S1. Patients were classified as “lower-risk” (IPSS-R≤3) and “higher-risk” (IPSSR≥4.5). This study was approved by the Ethics Committee of the Sixth Hospital affiliated to Shanghai Jiao Tong University (Shanghai, China), and all patients’ relevant research strictly abided by the Declaration of Helsinki.

### Animals

NHD13 transgenic mice, Setd2 ^fl/fl^ mice and Lep-CreSetd2 ^fl/fl^ mice were obtained from CAS Key Laboratory of Tissue Microenvironment and Tumor, Shanghai Institute of Nutrition and Health, Shanghai Institutes for Biological Sciences, University of Chinese Academy of Sciences, Chinese Academy of Sciences (Shanghai, China). All mice used in the experiments were on a pure C57BL/6 genetic background. The mice were used according to animal care standards, and animal studies were approved by the Committee of Animal Use at Shanghai Institute of Nutrition and Health.

### Cell lines and cell culture

The cell line SKM-1 was maintained in RPMI-1640 medium (Laisi Biosciences Inc, Shanghai, China) supplemented with 10% FBS and 100 U/mL penicillin/streptomycin. The cell line K562 was maintained in IMDM modified (Laisi Biosciences Inc) supplemented with 10% FBS and 100 U/mL penicillin/streptomycin. Cells were maintained in a humidified atmosphere containing 5% CO _2_ at 37°C, and the culture medium was replaced three times per week.

### Reagents

RhS100a9 and Ultra-LEAF™ Purified anti-PD-1 were purchased from BioLegend (San Diego, USA). Primary antibodies were obtained from the following manufacturers: p-AKT and p-mTOR from Cell Signaling Biotechnology (Beverly, USA), p-PI3K and GAPDH from Beyotime Biotechnology (Shanghai, China), and CD41 and CD61 from Abcam (Cambridge, UK). HRP-conjugated secondary antibodies were purchased from Cell Signaling Biotechnology. Specifically, SKM-1 and K562 cells were stained with anti-PD-1-BV421 and anti-PD-L1-BV421 antibodies (BioLegend). Mouse peripheral blood was stained with anti-c-Kit-PE-Cy7, anti-TER-119-APC, anti-CD71-PE, anti-PD-1-APC and anti-PD-L1-PE antibodies (BioLegend).

### Cytotoxicity of lymphocytes

More than 2.8×10 ^6^ MDS-BM-MNCs were harvested via the density gradient centrifugation method through Ficoll (Lymphosep, Biosera, UK). After standing for 2 h, CD3 MicroBeads (Miltenyi Biotec, Köln, Germany) were used for lymphocyte selection. Suspension cells were collected, pretreated with 50 IU/mL IL-2 for 72 h, and then co-cultured with SKM-1 cells. After co-culture for 24 h, the cytotoxicity of lymphocytes was tested by CCK8 assay.

### Western blot analysis

Cells were lysed on ice for 10 min in RIPA buffer (Beyotime Biotechnology). The cell lysates were then centrifuged at 10,000 *g* , and the supernatants were collected for western blot analysis. Equal amounts of protein were separated by SDS-PAGE and blotted onto PVDF membranes. Membranes were incubated initially with primary antibodies and subsequently with corresponding secondary antibodies for 1 h. Specific bands were visualized using an enhanced chemiluminescence (ECL) western blot detection kit (Beyotime Biotechnology).

### Wright’s stain

Samples were fixed with reagent one (Nanjing Jiancheng Bioengineering Institute, Nanjing, China) for 1 min, and then stained with reagent two (Nanjing Jiancheng Bioengineering Institute; reagent one: reagent two=1:2) for 8 min. Samples were washed with water and then examined under a microscope after samples were air-dried.

### Hematoxylin and eosin staining

Tissue samples from the mouse bone marrow and spleen were separated, fixed in 4% paraformaldehyde, embedded in paraffin, and sectioned (5 μm thickness) using a microtome. Subsequently, the sectioned tissues were stained with hematoxylin and eosin (H&E), after which they were observed under a light microscope.

### Immunohistochemistry assay

Tissue samples from the mouse spleen were separated, fixed in 4% paraformaldehyde, embedded in paraffin, and sectioned (5 μm thickness) using a microtome. Subsequently, the sectioned tissues were incubated initially with primary antibodies and subsequently with corresponding secondary antibodies for 1 h. Brown particles were formed using a DAB Horseradish Peroxidase Color Development kit (Beyotime Biotechnology).

### Statistical analysis

All experiments were repeated at least three times. The results of multiple experiments are presented as the mean±standard deviation (SD). Statistical analyses were performed using GraphPad Prism version 8.0. *P* values were calculated utilizing Student’s *t*-test or one-way analysis of variance (ANOVA). *P*<0.05 was considered statistically significant.

## Results

### S100a9 induces cell apoptosis in MDS clone cells

S100a8/a9 elicits powerful anti-tumor responses and induces cell death program or apoptosis, which appears to be independent of death receptors like the Receptor of Advanced Glycation Endproducts or Fas-associated with death domain protein [2]. To identify the potential apoptosis-inducing effect of S100a9 on MDS clone cells, we tested the toxicity of S100A9 in human MDS cell lines SKM-1 and K562.

**Figure 1:**
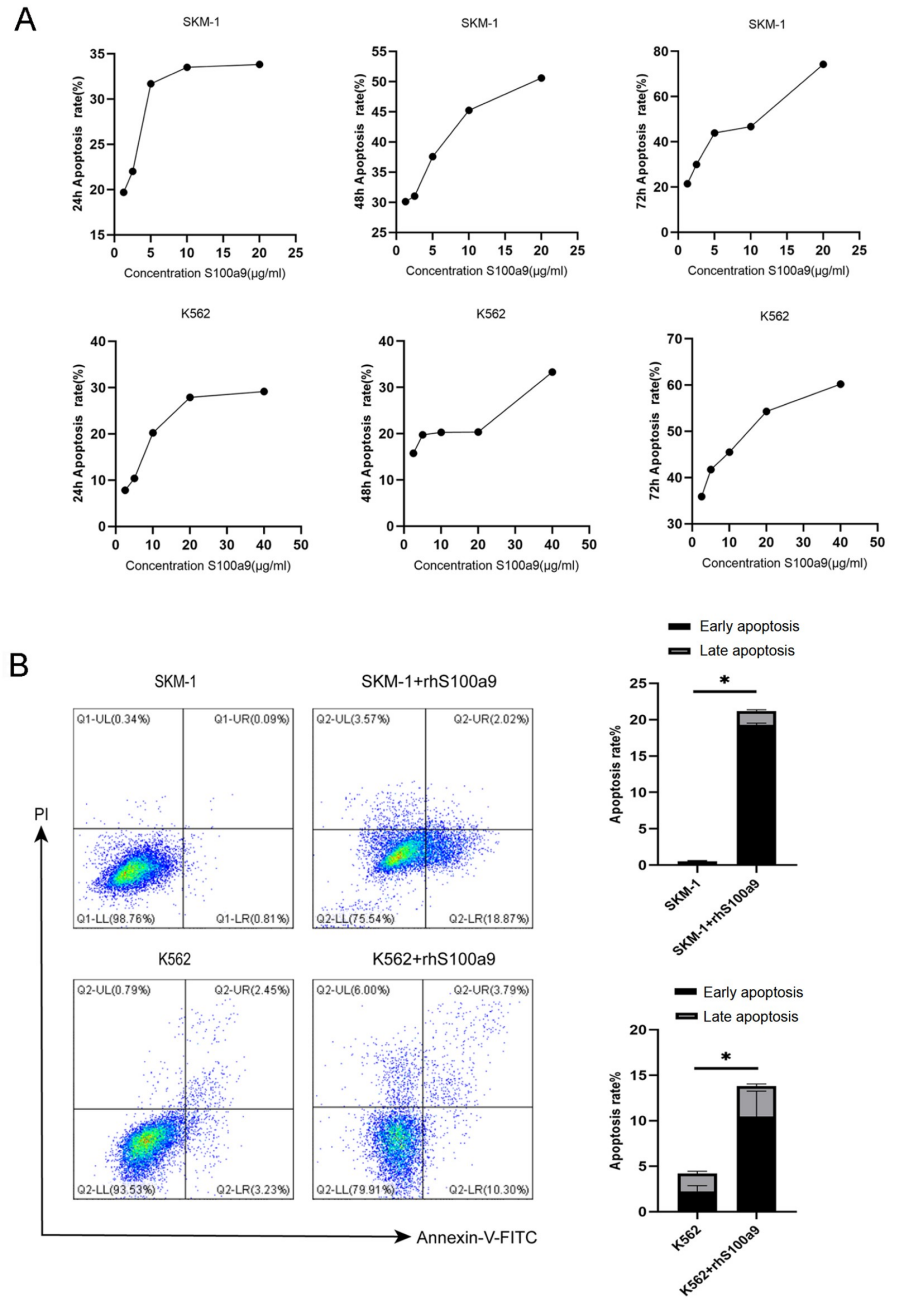
Figure 1 S100a9 induces cel apoptosis in MDS clone cells (A) The cytotoxicity of SKM-1 and K562 cells treated with rhS100a9 at different concentrations for 24, 48 and 72 h was tested by CCK8 assays. (B) Cells were treated with rhS100a9 (20 μg/mL for SKM-1, 40 μg/mL for K562) for 72 h. The early and late apoptosis rates were measured by flow cytometry. Data are expressed as the mean±SD from more than three experiments. * P<0.05.

First, we measured the S100a9 cytotoxicity levels in SKM-1 and K562 cells by CCK8 assay and found that cytotoxicity was higher in SKM-1 cells than in K562 cells at the same S100a9 concentration ( Figure 1A). PI and Annexin-V were used to determine cell apoptosis in SKM-1 cells and K562 cells ( Figure 1B). To further ascertain the role of S100a9 in MDS clone cells apoptosis, 20 μg/mL rhS100a9 was administered to SKM-1 cells and 40 μg/mL rhS100a9 was administered to K562 cells. It was found that the early and late apoptosis levels were increased as revealed by flow cytometry. The levels of early and late apoptosis were significantly increased after treatment with rhS100a9 ( Figure 1B).

### S100a9 rescues exhausted CD8 ^+^ T cells via the PI3K/AKT/mTOR signaling pathway

**Figure 2:**
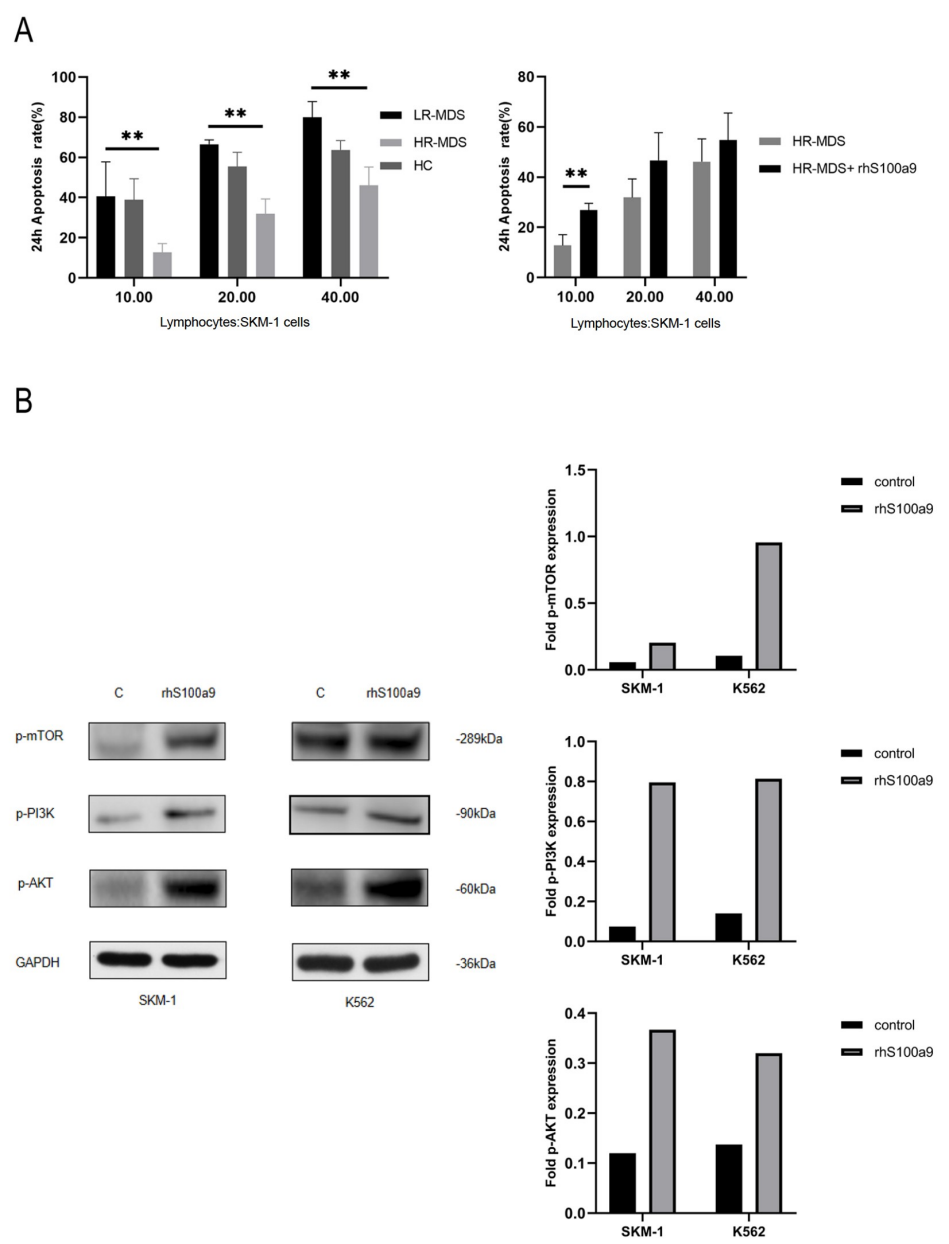
Figure 2 **S100a9 rescues exhausted CD8 ^+^** **T cells via the PI3K/AKT/mTOR signaling pathway** (A) Lymphocytes were isolated from MDS patient specimens and healthy control (HC) specimens ( n=4 LR-MDS, n=5 HR-MDS, n=3 HC and n=5 HR-MDS+rhsS100a9). Lymphocytes were treated with IL-2 (50 IU/mL) for 72 h and then cocultured with SKM-1 cells for 24 h. The cytotoxicity was measured by CCK8 assay. (B) SKM-1 cells and K562 cells were treated with rhS100a9 (20 μg/mL for SKM-1, 40 μg/mL for K562) for 72 h. PI3K, AKT and mTOR levels were measured by western blot analysis. Data are expressed as the mean±SD from three experiments. ** P<0.01.

It has been reported that the activation of PI3K/AKT enhances the nutritional intake and energy production of CD8 ^+^ T cells, and mTOR is responsible for regulating the biological effects of immune cell stimulation [13]. To determine whether S100a9 is responsible for the cellular cytotoxicity of lymphocytes, lymphocytes and SKM-1 cells were mixed and co-cultured at a certain ratio with or without S100a9 for 24 h. Then the cytotoxicity of lymphocytes was tested by CCK8 assay. Cytotoxicity was higher in lower-risk MDS-lymphocytes than in high-risk MDS-lymphocytes, and S100a9 partially rescued the exhausted cytotoxicity in lymphocytes ( Figure 2A). We also detected PI3K/AKT/mTOR protein levels by western blot analysis. The levels of PI3K, AKT and mTOR were significantly increased after treatment with rhS100a9 ( Figure 2B).

### PD-1/PD-L1 is involved in S100a9-induced tumor escape

**Figure 3:**
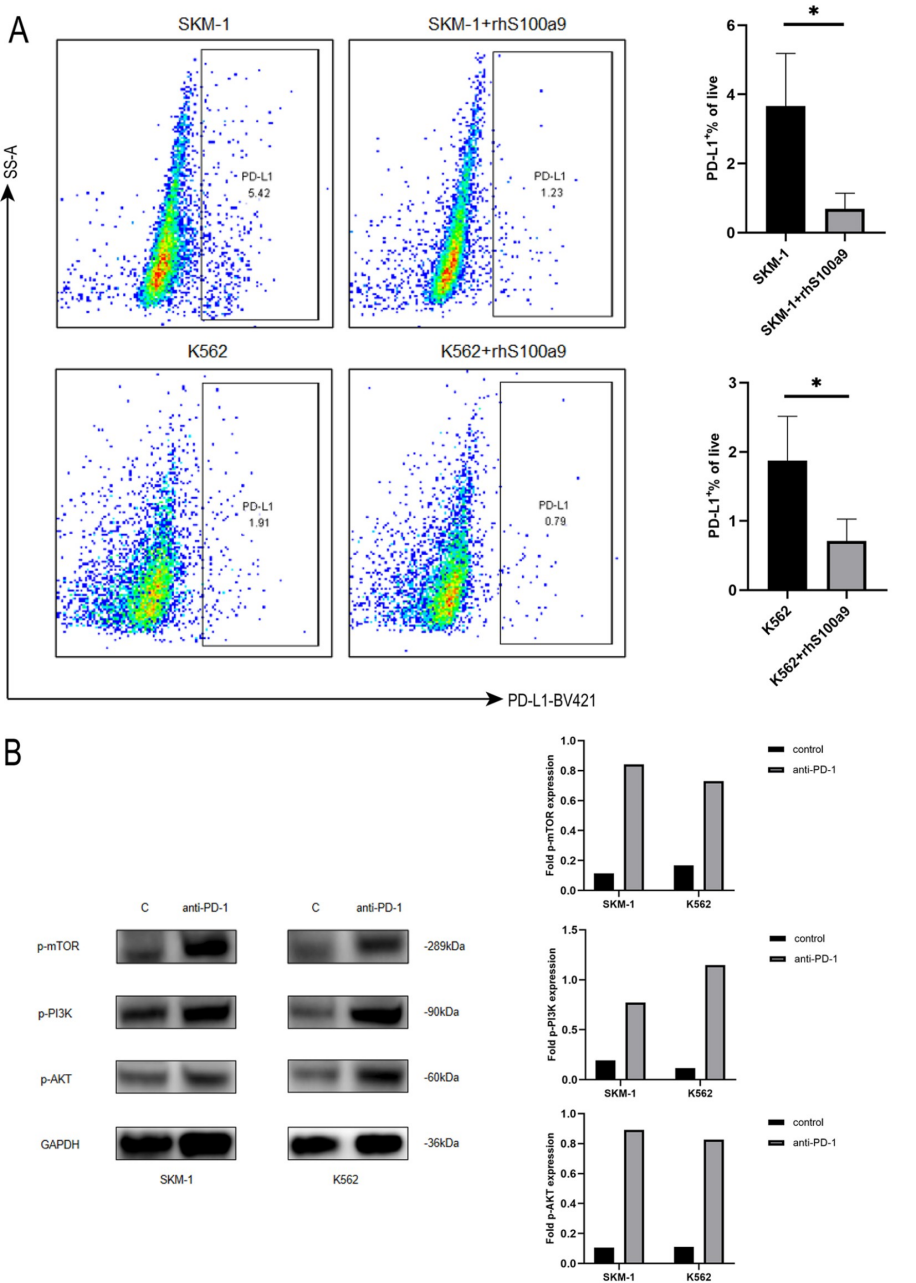
Figure 3 PD-1/PD-L1 is involved in S100a9-induced tumor escape (A) SKM-1 cells and K562 cells were treated with rhS100a9 for 72 h. PD-L1 level was measured by flow cytometry. (B) Western blot analysis of the expressions of PI3K, AKT, and mTOR in SKM-1 and K562 cells treated with anti-PD-1 (5 μg/mL). Data are expressed as the mean±SD from three experiments. * P<0.05.

We have previously demonstrated that S100a9 is highly expressed in lower-risk MDS-BM-MNCs and expressed at low levels in higher-risk MDS-BM-MNCs [4]. Cheng *et al*. [11] reported that the expressions of PD-1 on HSPCs and PD-L1 on MDSCs were increased in MDS versus in healthy donors. In addition, PD-1/PD-L1 blockade rescued exhausted CD8 ^+^ T cells via the PI3K/AKT/mTOR signal pathway [14]. Thus, we speculated that S100a9 may rescue exhausted CD8 ^+^ T cells via PD-1/PD-L1 blockade and possibly also elicit powerful anti-tumor responses. The results showed that the levels of PD-1/PD-L1 were markedly decreased in SKM-1 and K562 cells after treatment with S100a9 ( Figure 3A). Furthermore, we detected the expressions of PI3K, AKT and mTOR in SKM-1 and K562 cells treated with an anti-PD-1 blocking antibody (5 μg/mL) by western blot analysis. Consistent with our previous results, PI3K, AKT and mTOR levels were higher after treatment with the anti-PD-1 blocking antibody ( Figure 3B).

### PD-L1 is increased in (NHD13)Setd2 ^fl/fl^ transgenic and (NHD13)Lep-creSetd2 ^fl/fl^ transgenic mice

**Figure 4:**
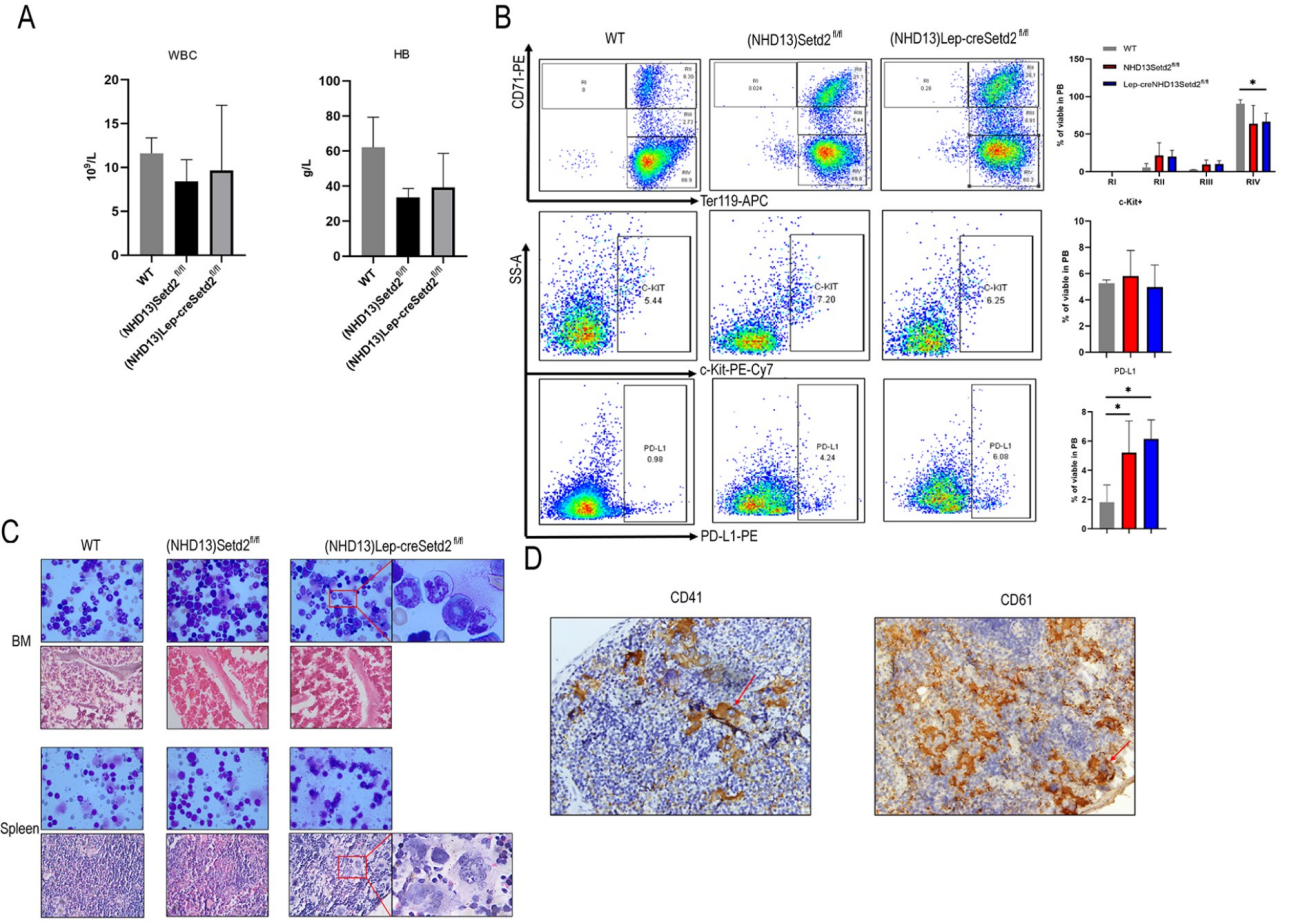
Figure 4 **PD-L1 is increased in (NHD13)Setd2 ^fl/fl^** **transgenic and (NHD13)Lep-creSetd2 ^fl/fl^** **transgenic mice** (A) Complete blood count (CBC) analysis of WT, (NHD13)Setd2 fl/fl and (NHD13)Lep-creSetd2 fl/fl mice. CBCs were obtained 3 months after transplantation. RBC, hemoglobin. (B) Representative flow cytometry profiles and quantification of the frequencies of the peripheral blood (PB) cells of the WT, (NHD13)Setd2 fl/fl and (NHD13)Lep-creSetd2 fl/fl mice at the indicated erythroid differentiation stages (RI, proerythroblasts; RII, basophilic erythroblasts; RII, chromatophilic erythroblasts; RIV, orthochromatophilic erythroblasts). Representative flow cytometry profiles and quantification of the frequencies of c-kit+ cells in the PB of the indicated mice at 3 months after transplantation. Representative flow cytometry profiles and quantification of the frequencies of PD-L1+ cells in the PB of the indicated mice at 3 months after transplantation. (C) Wright’s staining of BM and spleen cells isolated from mice transplanted for 3 months (100×magnification). Hematoxylin and eosin staining of the BM and spleen of the mice transplanted for 3 months (40×magnification). (D) Immunohistochemistry of spleen of the (NHD13)Lep-creSetd2 fl/fl mice transplanted for 3 months (40×magnification) in CD41 and CD61. Data are expressed as the mean±SD from three experiments. * P<0.05.

To examine PD-L1 expression in MDS mice, we performed BM transplantation by transplanting NHD13-expressing WT or Setd2 ^fl/fl^ BM cells into lethally irradiated recipient mice (Setd2 ^fl/fl^ mice and Lep-creSetd2 ^fl/fl^ mice). We found that, although with no statistical significance, the (NHD13)Setd2 ^fl/fl^ group and (NHD13)Lep-creSetd2 ^fl/fl^ group had trends toward lower WBC and lower hemoglobin expression compared with the WT group ( Figure 4A).

We examined erythroid differentiation in the peripheral blood (PB) of WT, (NHD13)Setd2 ^fl/fl^, and (NHD13)Lep-creSetd2 ^fl/fl^ mice. Flow cytometry analysis of cells double stained with Ter119 and CD71 was used to distinguish different erythroid developmental stages, including proerythroblasts, basophilic erythroblasts, chromatophilic erythroblasts, and orthochromatophilic erythroblasts. We observed altered erythroid differentiation stages with decreased frequencies of mature erythroid cells in the PB of the (NHD13)Setd2 ^fl/fl^ mice and (NHD13)Lep-creSetd2 ^fl/fl^ mice compared with the WT mice ( Figure 4B). To examine MDS progression to AML, we tested representative flow cytometry profiles and quantified the frequencies of c-kit+ cells in the PB of the indicated mice. All the groups had an average percentage of c-kit+ cells in the PB ( Figure 4B). We examined PD-L1 expression in MDS mice ( Figure 4B). The (NHD13)Setd2 ^fl/fl^ group and (NHD13)Lep-creSetd2 ^fl/fl^ group had significantly higher PD-L1 expression than the WT group, which verified the results in MDS cell lines. We also observed pathology in the BM and spleen of the three mouse groups ( Figure 4C). In Wright’s staining of BM, (NHD13)Lep-creSetd2 ^fl/fl^ mice significantly promoted aggressiveness of bone marrow hematopoiesis, such as annular nuclei and neutrophils lacking granules ( Figure 4C). Hematoxylin and eosin staining of the spleen showed that (NHD13)Lep-creSetd2 ^fl/fl^ mice significantly promoted the proliferation of megakaryocytes ( Figure 4C). Immunohistochemistry of the spleen of the (NHD13)Lep-creSetd2 ^fl/fl^ mice confirmed the promoted proliferation of megakaryocytes in hematoxylin and eosin staining ( Figure 4D).

## Discussion

MDS is mainly characterized by ineffective hematopoiesis, leading to peripheral cytopenias and progressive bone marrow failure [15]. The pathogenesis of this disease likely depends on the interaction between aberrant hematopoietic cells and their microenvironment. Furthermore, chronic inflammatory stimulation, in combination with senescence-dependent changes, in both hematopoietic stem/progenitor cells and the bone marrow microenvironment is believed to be critical to the pathogenesis of the disease.

Pro-inflammatory signaling and immune dysfunction have been identified as key pathogenic drivers of MDS [16]. In tumors, PD-1 has been shown to be expressed on exhausted T cells and to be a major mechanism of immune escape. Our previous studies showed that S100a9 expression was higher in low-risk MDS and lower in high-risk MDS. In this study, we revealed that PD-L1 is highly expressed in high-risk MDS. Furthermore, SKM-1 cells and K562 cells cocultured with S100A9 acquired apoptotic features, as evidenced by increased early and late apoptosis. S100a9 secreted from tumor cells is an important regulator of the tumor microenvironment and is an endogenous inhibitor of PD-1/PD-L1. Our study confirmed the inhibitory effect of S100a9 on PD-1/PD-L1.

The activated PI3K/AKT/mTOR pathway can improve T lymphocyte metabolism, nutrient uptake and energy production, regulate cell cycle and apoptosis, and affect T lymphocyte activation and immune function [ 17‒ 20] . Engagement of PD-L1 with the PD-1 receptor on T cells results in decreased effector T-cell function and apoptosis of T cells [ 21‒ 23] . In this study, we found that PD-1/PD-L1 blockade and S100A9 treatment both activated the PI3K/AKT/mTOR signaling pathway. The cytotoxic effects of S100a9 on cells and its cell death-inducing effects occur in a time- and concentration-dependent manner. Cells treated with high concentrations of S100a9 caused apoptosis, and low concentrations caused tumor invasion. Actually, this is in line with MDS. S100a9 was highly expressed in lower-risk MDS, which presented as ineffective hematopoiesis, leading to peripheral cytopenia, and expressed at low levels in higher-risk MDS, which was characterized by AML progression. Therefore, in higher-risk MDS, treatment with a high concentration of S100a9 partially rescues the exhausted cytotoxicity in lymphocytes. Therefore, we concluded that S100a9 may inhibit MDS-associated tumor escape via PD-1/PD-L1 blockade through PI3K/Akt/mTOR signaling pathway activation.

In a recent multicohort study, the tolerable safety profile and positive BM response suggest a beneficial use of anti-PD-1 agents in the treatment of MDS [24]. Treatment of MDS clone cells with AZA resulted in a dose-dependent upregulation of PD-1/PD-L1. Exposure to AZA resulted in partial demethylation of PD-1 in MDS cell lines and human samples [25]. Therefore, combination therapy with AZA and nivolumab has better efficacy than monotherapy with AZA [26]. However, PD-1 inhibition alone did not seem to have any beneficial impact on disease outcomes, and we cannot predict the efficacy accurately when using anti-PD-1 agents in MDS patients.

Our results are consistent with results in clinical trials. The experimental results indicated that the possible mechanisms by which anti-PD-1 agents may contribute to the treatment of MDS. These insights may provide mutation-specific treatment as a supplementary therapy for MDS patients with high-risk mutations, such as TP53, N-RAS and other complex mutations. Although these mutations cannot be overcome by allogeneic stem cell transplantation, we may improve the microenvironment in these patients through anti-PD-1 agents or other immune-modulatory agents. Then, we can provide more accurate new drug combinations and validate the feasibility of the mechanism.

## Supporting information

210SupplementaryMaterials

Supplementary

## Supplementary Data

Supplementary data is available at *Acta Biochimica et Biophysica Sinica* online.
