# Supplementary material for: S100a9 deficiency accelerates MDS-associated tumor escape via PD-1/PD-L1 overexpression: S100a9 deficiency accelerates tumor escape
Source: Acta Biochim Biophys Sin (Shanghai). 2023 Feb 21;55(2):194–201. doi: 10.3724/abbs.2023015 (PMC10157523; doi:10.3724/abbs.2023015)
Supplement: 210SupplementaryMaterials [file 210SupplementaryMaterials.pdf]

Supplementary Table S1. The characteristics of patients enrolled in this study

Parameter	Number
Sex	
Male	9
Female	8
Median age, years (range)	70 (35 − 79)
WHO classification	
RCUD	1
RARS	1
RCMD	2
RAEB-1	6
RAEB-2	4
IPSS-R	
≤3	4
≥4.5	10
Karyotype	
Normal	13
Complex	4
